# Understanding the barriers and improving care in type 2 diabetes: Brazilian perspective in time to do more in diabetes

**DOI:** 10.1186/s13098-017-0244-y

**Published:** 2017-06-15

**Authors:** Sérgio Vencio, Päivi M. Paldánius, Matthias Blüher, Daniel Giannella-Neto, Rafael Caiado-Vencio, W. David Strain

**Affiliations:** 1 Federal University of Goiás-Postgraduate Programme, Goiânia, Brazil; 2 Novartis Pharma AG, Basel, Switzerland; 3 Department of Medicine, University of Leipzig, Leipzig, Germany; 4 Uninove University, São Paulo, Brazil; 5 Catholic University of Goiás, Goiânia, Brazil; 6 Diabetes and Vascular Medicine, University of Exeter Medical School, Exeter, UK; 7 Avenida T-4 número 313 Setor Bueno, Goiânia, Goiás CEP 74884-582 Brazil

**Keywords:** Diabetes, Clinical inertia, Brazil

## Abstract

**Background:**

Type 2 diabetes mellitus (T2DM) is a complex disease, particularly in a continental country like Brazil. We attempted to understand and evaluate the perceptions and routines of Brazilians with T2DM and physicians, compared with other countries.

**Methods:**

We compared the results from a 20-min online survey in Brazil with simultaneously collated data from India, Japan, Spain, UK and USA.

**Results:**

In total, 652 adults with T2DM and 337 treating physicians were enrolled, of whom 100 patients and 55 physicians were from Brazil. The numbers of primary care physicians from the five countries were 221 versus 43 in Brazil, diabetes specialists were 61 versus 12. There was disconnect between the opinions of physicians and people with diabetes globally. Further, there were differences between clinical practices in Brazil versus the rest of the world, in many areas Brazilians were performing better.

**Conclusions:**

Communication between patients and physicians should be clearer. There is an urgent need to identify the deficits in education, in order to address the clinical inertia within the diabetes management team. There is a necessity to understand the specific requirements of the Brazilian population in order to contextualise international guidelines and implement local changes in practice.

## Background

According to International Diabetes Federation, approximately 14.3 million people are living with diabetes in Brazil, and this number is expected to increase to 23.3 million by the year 2040 [1]. Good glycaemic control soon after diagnosis, results in significant long-term benefits, reducing the prevalence of micro and macrovascular complications. These benefits persist for many years, indeed the glycaemic control at diagnosis affects outcomes many years later [2]. As diabetes progresses, the benefit of a good glycaemic control is attenuated and may even be paradoxically reversed [3].

Despite this knowledge, however, there remains a substantial gap between clinical aspirations and targets achieved [4]. A significant contributor to this gap is clinical inertia, that is, a delay in escalating therapy at the appropriate time. Clinical inertia has been widely recognised as a barrier in the management of diabetes for many years; however, the proportion of people with diabetes achieving blood pressure, lipid and glycaemic targets has not substantially improved [5]. Recently, the clinical consequences of inertia have also been associated with increased incidence of cardiovascular complications in subjects not achieving their glycaemic targets [6]. This is partially due to a lack of understanding of the factors that influence escalation of therapy. Most of the studies have explored the determinants of clinical inertia in USA, UK and few South-Asian countries [7–9], however, studies highlighting factors responsible for clinical inertia in Brazil are lacking even though the rate at which people being affected with diabetes is increasing alarmingly [1]. We conducted a survey (Time to do More) involving both people with diabetes and physicians across six countries in order to understand the potential causes of clinical inertia. The current subanalysis of the ‘Time to do More’ survey aimed to understand the perceptions and routines of patients with type 2 diabetes and physicians in Brazil in comparison to rest of the countries (USA, UK, Spain, India and Japan).

## Methods

Details of the Time to do More survey have been published elsewhere [10]. Briefly, individuals from the Kantar Health panel of over 2500 physicians and 118,000 patients who agreed to be contacted for research purposes were randomly invited for a 20-min online survey. After introduction of quotas, the survey enrolled 652 people with type 2 diabetes and 337 physicians (264 general practitioners [GPs]; 73 specialists) treating diabetes, from Brazil, Japan, India, Spain, UK and USA. Fifty-five Brazilian physicians and 100 Brazilian people with diabetes contributed to the final results (Table 1).

**Table 1:** Demographic characteristics of the patients and physicians

	USA (n = 151)	UK (n = 100)	Spain (n = 100)	India (n = 100)	Japan (n = 101)	Brazil (n = 100)
Male	58%	60%	60%	62%	72%	60%
Female	42%	40%	40%	38%	28%	40%
Mean age (in years)	60.6	59.6	53.3	52.3	57.8	52.4
Mean BMI (kg/m^2^)	33.1	31.0	28.9	24.7	24.9	33.9
Employed full-time	20%	26%	29%	53%	47%	48%
Employed part-time	7%	18%	13%	13%	7%	22%
Student	–	–	–	1%	–	3%
Not working for health reasons	17%	9%	7%	4%	3%	8%
Not working for other reasons	9%	1%	27%	4%	13%	2%
Retired	47%	46%	24%	25%	31%	17%
Low income	33%	46%	85%	31%	52%	8%
Middle income	48%	28%	3%	30%	35%	23%
High income	15%	10%	4%	33%	5%	66%
Prefer not to say	4%	16%	8%	6%	8%	3%

	USA (n = 75)	UK (n = 50)	Spain (n = 51)	India (n = 50)	Japan (n = 56)	Brazil (n = 55)
PCPs	80%	80%	80%	80%	71%	78%
Endocrinologist/diabetologist	20%	20%	20%	20%	29%	22%
Mean time as a medical doctor (in years)	18	19	17	14	24	15
Average proportion of time spent counselling patients	97%	87%	88%	81%	92%	91%
Average number of T2DM patients seen in a month	170	102	125	261	209	124

*BMI* body mass index, *PCPs* primary care physicians, *T2DM* type 2 diabetes mellitus

The survey was designed with the following objectives:

- To identify barriers in improving the treatment of type 2 diabetes mellitus (T2DM) and understand the ways in which these can be overcome.
- To understand clinical inertia and to what extent it constitutes a barrier to improving care in T2DM.
- To explore perceptions on treating earlier and more aggressively.
- To identify areas of unmet need.

Inclusion criteria are provided in Table 2.

**Table 2:** Inclusion criteria for physicians and patients

Physician screening criteria	Patient screening criteria
3–35 years in practice	Confirmed diagnosis of T2DM
Spend at least 70% of time in patient management Every month see at least 50 patients with T2DM (for PCPs), 100 patients for specialists Prescribes oral and/or injectableT2DM treatments	Quotas on age, gender, number of pills taken in a day, economic background (low, average, high income brackets)

*PCPs* primary care physicians, *T2DM* type 2 diabetes mellitus

Specific questions explored recollection from both physicians and people with diabetes on topics discussed at initial diagnosis consultation and follow-up consultations, attitudes towards complications, adequate disease management and perceived likelihood of achieving treatment targets. Physicians were also questioned regarding any modification to treatment algorithms for elderly patients (aged 80 years) or those with co-morbidities (as typified by renal impairment) compared to a typical 50-year old individual with diabetes. Responses from Brazil were compared against those pooled from Japan, India, Spain, UK and USA. Continuous data were used whenever possible to maximize power.

## Results

Overall, 100 people with diabetes from Brazil completed the survey, compared to 552 from the rest of the world. There were 43 PCPs from Brazil, compared to 221 in the global pool and 12 diabetes specialists compared with 61 from Japan, India, Spain, UK and USA.

### Initial consultation visit: diagnosis

Globally, 68% of participants with diabetes received diagnosis from a PCP, however, in Brazil the responsibility was reversed, such that 53% of participants recall their initial diabetes counselling coming from a specialist, with PCPs accounting for a further 34% and other specialists, particularly cardiologists and nurses confirming the diagnosis for the rest of the participants.

This involvement with diabetes specialists at diagnosis translated into longer initial consultations, with a quarter of Brazilians being offered >40 min, and only 17% receiving <20 min. This compares very favourably with figures worldwide, where 74% of initial consultations lasted <20 min and a further 19% of people only receiving between 20 and 40 min.

People with diabetes in Brazil had similar perception about their initial consultation, with 72% recalling longer than 20 min and 36% longer than 30 min. However, globally, only 61% people with diabetes recalled receiving consultation for at least 20 min.

### Topics discussed during initial consultation

**Fig. 1:**
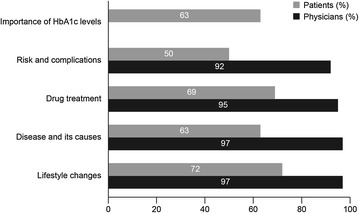
Respondents (physicians [n = 337], patients [n = 652]) recall of the topics discussed at the diagnosis consultation. *HbA1c* glycosylated haemoglobin

Lifestyle changes, disease and its causes and drug treatment were topics discussed by almost all physicians at the diagnosis consultation; however, fewer patients recalled these topics being discussed (Fig. 1).

There were no important differences across countries in topics that were discussed at the diagnosis consultation or in the recollection of these topics. Lifestyle changes, such as diet and exercise, were discussed by physicians of all countries in nearly all cases, and the importance of glycaemic targets was discussed in 85% of consultations in Brazil and 89% globally.

A vast majority of physicians discussed the disease and its causes, drug treatment and risks and complications of T2DM, whereas about 1 in 5 patients had no recollection of any of these. A similar number of patients recalled discussing lifestyle changes in Brazil (13%) as in the rest of the world (16%). The impact of other health conditions on the management of diabetes was discussed only in a small minority of patients globally (2%); this discussion was twice as likely to be had in Brazil (5%). However, it is impossible to determine whether it was due to a higher prevalence of co-morbidities at diagnosis in Brazilian people with diabetes compared with the rest of the world. Fewer people from Brazil recalled the discussion about risks and complications of T2DM (6%) compared with the other countries (9%).

**Fig. 2:**
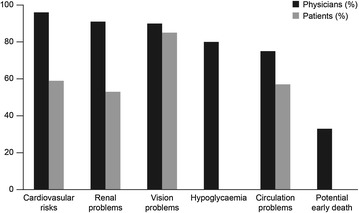
Recollection of the risks and complications discussed during diagnosis consultation by physicians (n = 337) and patients (n = 206)

The longer duration of initial consultation was associated with fewer people with diabetes having difficulty in understanding the topics discussed. Managing the disease during fasting periods was considered difficult to understand for 35% globally versus 20% in Brazil. Similarly, less than half of people with diabetes in Brazil struggled to understand the costs associated with treating diabetes compared with the rest of the world (10% vs 25% respectively), and the potential side effects of this treatment caused fewer concerns amongst Brazilians with only 12% reporting unease at treatment compared with 20% globally (Fig. 2).

Patients were overall satisfied with the diagnosis consultation duration, and to a great extent seemed to understand the key topics of lifestyle changes, disease and its causes and drug treatment. Most of them seemed to accept the T2DM in a positive way.

Understanding of T2DM complications was slightly more limited, and there was a high level of disconnect between what physicians thought they had discussed and what patients were able to recall.

### Follow-up visits

Follow-up visits occurred fairly regularly and lasted for 15 min on an average, with approximately 5 visits per year. Only a minority of people with diabetes stated they would like to see the physician more often. Interestingly, this was not associated with the actual frequency of visits. For example, the inter-visit length in Brazil for patients on diet and exercise treatment alone was approximately 12 weeks contrasting with 17.2 weeks globally; however, 1 in 4 Brazilian patients considered visits were very infrequent.

Patients on oral anti-hyperglycaemic agents or insulin had the same average frequency of visits. The follow-up visits were longer in Brazil (25 min) compared with the rest of the world (13.4 min). As expected, most of the consultation was spent on taking history and diagnosis tests to check if the disease was under control.

### Discussing complications

Majority of physicians believed they had adequately explained T2DM complications to their patients, including approximately a third explaining the risk of potential early death. However, only a quarter of patients reported that they were worried about developing these complications of T2DM, while the rest were either not concerned or thought the risk was remote.

The complication that concerned patients the most was the potential of vision being affected or complications leading to blindness, with half of people reporting they were worried about this risk, while 21% reporting concern regarding cardiovascular disease. A higher proportion of Brazilian physicians recalled discussing the importance of complications, such as circulation problems (89% vs 71%) and potential early death (44% vs 30%), to their patients. Paradoxically, fewer Brazilian physicians spent time explaining the potential impact of the disease on sexual health and fertility compared to the rest of the world (49% vs 65%).

Regarding patients’ recall memory, more international patients recalled the discussion about potential kidney problems (59% vs 39%).

The risk of developing complications had a more profound effect on Brazilians; 39% were devastated to hear they might develop complications versus 25% in the rest of the world.

Only 3% of Brazilian patients were not concerned over the risk of developing complications compared with 10.8% globally, which was again proportionate to the increased time spent at the initial consultation. This concern also perpetuated into follow-up visits, where nearly 50% more Brazilians remained concerned through follow-up visits than in the rest of the world (72% vs 50% respectively). Despite this, apprehension over the need of future injectable therapy was a greater concern in Brazil than globally, both at diagnosis (59% vs 47%) and at follow-up visits (51% vs 38%).

Fewer Brazilian physicians believed their patients did not understand the serious consequences of hypoglycaemia and the importance of reporting events (58% vs 67%). Concordantly, Brazilian people with diabetes were less likely to know very little or nothing about hypos compared with the rest of the world (11% vs 21%). Further, Brazilians were more aware of the link between hypoglycaemia and premature mortality compared with other countries (13% vs 4.4%).

### Treatment algorithm

More than half of the physicians globally (53.2%) agreed that early treatment using combination therapy could help control blood sugar levels and reduce risk of complications compared with only 25% in Brazil. Consequently, use of combination therapy as first line was very rare, with most physicians reserving this powerful approach for second line.

Despite acknowledgement that there were important differences in the needs of elderly patients or those with co-morbidities (as represented here by people with renal failure), initial treatment algorithms were remarkably similar, with only 7% physicians stating they would initiate combination therapy at diagnosis in patients diagnosed at 50 years, 9% for patients aged >80 and 7% for people with renal impairment. Interestingly, in this latter group, Brazilian physicians were more likely to recommend insulin at diagnosis compared with the rest of the world (34% vs 21.4%, respectively).

Approximately a quarter of people with a new diagnosis of diabetes did not receive drug therapy at their initial consultation (25% in Brazil and 23% globally); however, nearly half of Brazilian physicians had initiated a drug within a month, compared with only a third in the rest of the world.

When questioned about their glycosylated haemoglobin (HbA1c), the majority of people with diabetes in all countries reported an HbA1c between 6.5 and 7.5%; however, a third of participants in Spain and Brazil did not know their HbA1c levels (Table 3).

**Table 3:** Average blood sugar levels (HbA1c) reported by the patients during survey

Average blood sugar/HbA1c levels	USA (n = 151)	UK (n = 100)	India (n = 100)	Japan (n = 101)	Spain (n = 100)	All five countries (n = 552)	Brazil (n = 100)
≤6.5%	28	20	14	39	18	24	5
>6.5 and ≤7.0%	21	18	31	23	12	21	25
>7.0 and ≤7.5%	16	18	25	15	17	18	23
>7.5 and ≤8.0%	5	10	9	7	10	8	6
>8.0 and ≤8.5%	3	3	–	5	2	3	4
>8.5%	1	5	2	3	6	3	4
I don’t know	26	26	19	9	35	23	33

Values are represented as percentages

*HbA1c* glycosylated haemoglobin

When questioned about the willingness to comply with lifestyle advice, approximately half of patients reported no intention of changing their diet, and three-fifth did not intend to exercise more. Reasons for this willful non-compliance were very similar globally. Interestingly, proportion of those reporting financial concerns as a justification for the lack of compliance was almost identical in Brazil compared with the rest of the world (19 and 18%).

## Potential for improvement

Over half of physicians in Brazil and the rest of the world reported the desire for more time to discuss disease management to achieve better adherence to therapies, lifestyle changes, obesity management and early screening (Brazil 55 and 56% globally).

Better treatment options was second on physicians’ wish list. This was particularly pertinent amongst Brazilian physicians who desired more effective treatments, easier drug regimen, less frequent administration and medications with fewer side effects/lower risk of hypos (40% vs 24%). Inconsistently, Brazilian physicians were half as likely to request more patient education programs compared to international physicians (7% vs 17%).

## Discussion

The Time to do More in diabetes survey demonstrated a clear disconnect in communication between healthcare providers and people with diabetes [10]. We have demonstrated that by allowing more time for patients in Brazil and having a greater involvement of specialists at an early stage, people with T2DM in Brazil have a good understanding of the risk of hypoglycaemia and increased awareness of the risks of poorly controlled diabetes compared with the rest of the world. Notwithstanding engagement in the basic interventions such as diet and lifestyle remained low, albeit better in the Brazilian population than in the rest of the world.

In 2013, a National Health Survey in Brazil, showed that 6.9% of the population aged ≥18 years self-reported diabetes mellitus. There was a higher rate of diagnosis of diabetes (9.6%) among individuals with no education or with incomplete elementary school. Rates ranged from 0.6% (age group 18–29 years) to 19.9% (age group 65–74 years). There was no statistically significant difference in the diagnosis rates among whites, blacks and mixed race individuals [11].

At diagnosis, a quarter of consultations lasted over 41 min, substantially more than that noted globally; however, this did not necessarily translate to increased patient satisfaction, with half of Brazilian patients believing that they did not have time to explain their fears and concerns. Diet and exercise were discussed at length by the physicians, but only two-thirds of the patients understood the importance of these lifestyle interventions.

The current Brazilian guidelines, in an attempt to combat clinical inertia, acknowledge that monotherapy is unlikely to achieve glycaemic control if HbA1c at diagnosis is >7.5% or fasting glucose is >200 mg/dL [12]. Therefore, the guidelines recommend combination therapy at diagnosis in patients with high baseline HbA1c. The use of combination therapy at diagnosis could positively influence the durability of glycaemic control, changes in insulin sensitivity and β-cell function, time to insulin introduction, influences diabetic complications and the effects on some surrogates [13].

Three of the most important medical societies in Brazil (diabetes, endocrinology and cardiology) have teamed up to set a multidisciplinary management diabetes guideline. One important statement was that fighting clinical inertia, defined as delay in treatment schedule at the appropriate time, should be effective for glycaemic targets, blood pressure and lipid (unpublished data). However, in our survey, only 25% of Brazilian physicians indicated they would initiate combination therapy at outset. This figure was similar to that reported globally, suggesting there is a global inertia to commencing combination therapy, while the physicians acknowledged the scientific evidence behind the introduction of early combination.

This is an interesting paradox of diabetes management—a cardiologist would not hesitate to add multiple anti-platelet agents, anti-hypertensives and statin therapy at the diagnosis of myocardial infarction; a respiratory physician would routinely initiate combination of long-acting β-2 agonist and steroid therapy if the patient was sufficiently compromised, yet despite established evidence that a single oral agent is very unlikely to achieve good glycaemic control and address the key pathophysiological features of diabetes, most diabetologists will nevertheless implement a sub-optimal “treat-to-fail” strategy. Substantial training and re-education will be required in order to achieve adoption of current national and international guidelines to implement combination therapy at diagnosis.

Brazilian physicians do escalate care quickly than the rest of the world, with one-third more Brazilian patients receiving treatment shortly after diagnosis. This might reflect an acknowledgement of Brazilian Diabetes Society’s guidelines, suggesting rapid escalation of treatment until fasting glucose <100 mg/dL, pre-prandial glycaemia <130 mg/dL, postprandial glycaemia ≤160 mg/dL, and HbA1c <7% to avoid clinical inertia [12].

Importantly, there is a significant disconnect between what physicians say they have discussed with patients and what patients actually remember. Physicians wish to discuss issues of scientific importance; however, this often does not answer patients’ beliefs, fears and apprehensions.

Therefore, an important conclusion of this survey is that physicians should spend a long time determining the concerns, uncertainties and hesitations of people with diabetes before trying to impart their concern for their patients upon them.

The increased time spent at diagnosis consultation among the Brazilian patients, however resulted in a greater awareness of the risk of cardiovascular disease from the sum of multiple risk factors, and addressing these risk factors is very important for the prevention of up to two-third of deaths among people with diabetes [14].

At the time of the diagnosis, most of the people with diabetes were scared and insecure. Consequently, it is important to completely reassure the patients that with good glycaemic, lipid and blood pressure control, a normal quality of life and longevity can be achieved. Determining strategies to achieve good glycaemic control, including lifestyle changes, physical activity, healthy nutrition, self-monitoring, medical treatment compliance, consultation regularity and family involvement should become a priority.

Another advantage of the longer time with the physician at diagnosis was a greater, albeit low understanding of the importance of lifestyle changes. Brazilians recall 6% of the diagnosis consultation time spent discussing risks and complications of T2DM, compared with 9% globally.

The difference between the complications of greatest concern between the physicians and people with diabetes is interesting. Physicians were predominantly interested in complications with the highest mortality, with cardiovascular disease being of greatest concern. In contrast, people with diabetes, were far more interested in complications with an impact on the quality of life, notably retinopathy and nephropathy which is of major importance in clinical practice.

These microvascular complications, of greatest concern to our patients, have been demonstrated to be reduced by lifestyle changes including diet and exercise [15] and good metabolic control [16, 17]. If physicians focused on these quality of life-based discussions of complications rather than mortality-based discussions it addresses the greatest concern of patients, thereby improving engagement. Further, emphasis on treatable complications may invoke better commitment to diet, exercise and pharmacological therapy regimens.

This commitment may reap rewards in other areas, notably the risk of ulcer, amputation and severe neuropathy, in the diabetic foot. A recent multicentre study of 1455 people with diabetes in Brazil suggested a higher proportion of patients with neuropathic disease, predominantly neuroischaemic in nature, and a smaller number of patients with isolated ischaemic disease [18], compared to similar studies performed in the USA [19] and Western Europe [20].

Likewise, the prevalence of erectile dysfunction is higher among Brazilians than Western Europeans, with up to 74.6% of men with T2DM being affected, as assessed by international index of erectile function (IIEF-5) score [21]. In our study, the potential impact of diabetes on sexual health and fertility was given substantially less time in Brazil, with only 49% of physicians including it in their overview of potential complications compared to 64.6% in the other countries surveyed. At best, this indicates that patients may not completely be aware of the risks and potential treatments, and at worst, this lack of understanding could be contributing to the high-risk prevalence in Brazilian men and reduced quality of life.

## Study limitations

Although every effort was made to ensure the generalisability of the populations sampled, by utilising an online survey with predefined quotas, we may have generated some selection bias towards participants who are more technologically aware, more so in the people with diabetes than the physicians. However, we are more likely to have over-recruited the motivated and more educated, thus suggesting the true dissociation between physician and people with diabetes may be greater than represented here.

The use of the online questionnaire, however, is justified because it, for the first time, allowed survey of a large number of people with diabetes across six countries, specifically chosen to represent the different health care structures, from the entirely free at the point of delivery NHS service in the UK to the entirely private sector in the USA.

## Conclusions

Clinical inertia represents a considerable barrier to optimal diabetes treatment. A greater understanding of specific impediments to escalating care, particularly from the interactions between physicians and people with diabetes in Brazil, may help identify and therefore address the unmet needs of the population. Brazil offers more time than many other countries around the world to its newly diagnosed people with diabetes. However, we have highlighted that our principle concerns as health care providers, notably on preventing major cardiovascular events, are not the same as the priorities of our patients, who are more concerned about the microvascular complications that will impact quality rather than quantity of life. Adapting our consultation strategies to incorporate these patient-focused topics may improve understanding of the condition and ultimately engagement in self-management of the disease.

## Abbreviations

BMI: body mass index
GP: general practitioner
HbA1c: glycosylated haemoglobin
IIEP: international index of erectile function
NHS: National Health Service
PCPs: primary care physicians
T2DM: type 2 diabetes mellitus
